# Isopropyl Caffeate: A Caffeic Acid Derivative—Antioxidant Potential and Toxicity

**DOI:** 10.1155/2018/6179427

**Published:** 2018-04-19

**Authors:** Andressa Brito Lira, Camila de Albuquerque Montenegro, Kardilandia Mendes de Oliveira, Abrahão Alves de Oliveira Filho, Alexandre Rolim da Paz, Marianna Oliveira de Araújo, Damião Pergentino de Sousa, Cynthia Layse Ferreira de Almeida, Teresinha Gonçalves da Silva, Caliandra Maria Bezerra Luna Lima, Margareth de Fátima Formiga Melo Diniz, Hilzeth de Luna Freire Pessôa

**Affiliations:** ^1^Program of Postgraduate Studies in Natural Products and Synthetic Bioactive, Federal University of Paraíba, João Pessoa, PB, Brazil; ^2^Academic Unit of Health, Health and Education Center, Federal University of Campina Grande, Cuité, PB, Brazil; ^3^Program of Postgraduate Studies in Development and Technological Innovation in Medicine, Federal University of Paraíba, João Pessoa, PB, Brazil; ^4^Academic Biological Science Unit, Health Center and Rural Technology, Federal University of Campina Grande, Patos, PB, Brazil; ^5^Center for Medical Sciences, Federal University of Paraíba, João Pessoa, PB, Brazil; ^6^Department of Pharmaceutical Sciences, Federal University of Paraíba, João Pessoa, PB, Brazil; ^7^Postgraduate Program of Pharmaceutical Sciences, Federal University of Pernambuco, Recife, PE, Brazil; ^8^Department of Antibiotics, Federal University of Pernambuco, Recife, PE, Brazil; ^9^Department of Physiology and Pathology, Federal University of Paraíba, João Pessoa, PB, Brazil; ^10^Department of Molecular Biology, Federal University of Paraíba, João Pessoa, PB, Brazil

## Abstract

Phenolic compounds, among them isopropyl caffeate, possess antioxidant potential, but not without toxicity and/or adverse effects. The present study aimed to evaluate the antioxidant activity and toxicity of isopropyl caffeate through in silico, *in vitro* and *in vivo* testing. The results showed that isopropyl caffeate presents no significant theoretical risk of toxicity, with likely moderate bioactivity: GPCR binding, ion channel modulation, nuclear receptor binding, and enzyme inhibition. Isopropyl caffeate induced hemolysis only at the concentrations of 500 and 1000 *μ*g/ml. We observed types A and O erythrocyte protection from osmotic stress, no oxidation of erythrocytes, and even sequestrator and antioxidant behavior. However, moderate toxicity, according to the classification of GHS, was demonstrated through depressant effects on the central nervous system, though there was no influence on water and food consumption or on weight gain, and it did present possible hepatoprotection. We conclude that the effects induced by isopropyl caffeate are due to its antioxidant activity, capable of preventing production of free radicals and oxidative stress, a promising molecule with pharmacological potential.

## 1. Introduction

Phenolic compounds are widely distributed in plants; they protect against UV light, insects, bacteria, and viruses, all while inhibiting the growth of competitor plants [1, 2]. They are traditionally considered potent natural antioxidants because of their ability to eliminate hydroxyl radicals, aiding in the prevention of diseases associated with oxidative stress in membranes, proteins, and DNA [2–4].

**Figure 1:**
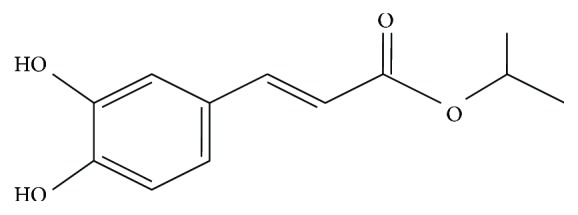
Isopropyl caffeate.

Isopropyl caffeate is a derived esterification of caffeic acid (Figure 1). This acid has low solubility, yet adding a chain alkyl to the molecule changes its lipophilicity, increasing permeability through the cell membrane and influencing its bioavailability [5, 6].

Isopropyl caffeate has also demonstrated various biological activities such as antinociceptive activity similar to that of licensed drugs [7], antioxidant activity according to the DPPH model [6], antitumor activity against different tumor strains [5, 6], activity significantly improving the *in vitro* inhibitory properties of renin [8], and antifungal effect against cellular and planktonic biofilm [9].

It is worth noting that no chemical is free of toxicity; the study of toxic effects is as necessary as knowledge concerning side effects, interactions, contraindications, and mutagenicity [10–12]. Substance toxicity is often related to the formation of free radicals, which cause oxidative stress and are responsible for a large number of diseases [13–15].

Oxidative stress is characterized as an imbalance between the production of reactive species and antioxidant defense activity [13–15]. It is noteworthy that free oxygen radicals involved in the etiology of inflammation cause damage to macromolecules, lipid peroxidation [16–18], and lead to cell apoptosis [17, 18].

In this context, our study examined isopropyl caffeate; initially using an in silico approach to characterize possible theoretical pharmacological and toxicological activities, we then investigated cytotoxicity and antioxidant effects, and finally, *in vivo* acute toxicity was determined.

## 2. Materials and Methods

### 2.1. General Procedure for the Synthesis of Isopropyl Caffeate

A mixture of caffeic acid (0.25 g) and isopropyl alcohol (50 ml) was heated under reflux in the presence of sulfuric acid (0.4 ml) until the completion of its reaction (8 hours), which was checked by a single spot in TLC. Then, alcohol was removed under reduced pressure and the solution was diluted with 20 ml of water. The product was extracted with ethyl acetate (15 ml). The organic phase was neutralized successively with 5% sodium bicarbonate and water, dried over anhydrous sodium sulfate, and filtered. After evaporation under reduced pressure, the phase yielded the ester derivative [19].

### 2.2. Isopropyl Caffeate

Black amorphous solid; yield 93.06%; Mp 155–160°C; IR ʋmax (KBr, cm^−1^): 3307, 3047, 2976, 1677, 1594 e 1442, 1277 e 1188. ^1^H NMR (DMSO-d6, 200 MHz): *δ*H 1.23 (6H; d; *J* = 6.2 Hz), 4.98 (1H; sept; *J* = 6.2 Hz), 6.23 (1H; d; *J* = 15.8 Hz), 6.75 (1H; d; *J* = 8.0 Hz), 6.99 (1H; dd; *J* = 8.0, 2.0 Hz), 7.04 (1H; d; *J* = 2.0 Hz), 7.44 (1H; d; *J* = 16.0 Hz), ^13^C NMR (CD3OD, 50 MHz): *δ*C 22.2, 69.0, 115.1, 115.6, 116.5, 122.9, 127.6, 146.6, 146.7, 149.4, and 168.8 [20]. The solubilization of the test product was performed with the aid of Cremophor RH 40 to 3% for the experimental protocols *in vitro*. For the *in vivo* protocol, Cremophor RH 40 to 10% was used.

### 2.3. Human Erythrocytes

Human erythrocytes of blood types A, B, O, and AB were obtained from bags containing erythrocyte concentrate, from the Transfusion Unit of the Lauro Wanderley University Hospital of UFPB. The project was approved by the Research Ethics Committee at the Lauro Wanderley University Hospital of UFPB, with approval number 1.658.669.

### 2.4. Animals

To perform the *in vivo* assay, *Mus musculus* mice, females, of the Balb/c strain from the Fiocruz Permanbuco Vivarium and weighing between 22–29 g were used. The mice were kept at a temperature of 21 ± 1°C, light and dark cycle of 12 h, and fed with pellet-type feed and water ad libitum. This experimental protocol was approved by the Committee on Ethics in Animal Use of UFPB, with registration number 009/2016.

### 2.5. In Silico Study: Molinspiration

The isopropyl caffeate was checked for bioactivity by calculating the activity score for each: GPCR ligand, ion channel modulator, kinase inhibitor, and nuclear receptor ligand. All of the parameters were checked with the help of the online Molinspiration software drug-likeness score. The drug-likeness score for each compound was calculated, compared with its specific activity, and the results were compared with the drug standard [21].

### 2.6. In Silico Study: PASS Online

Prediction of the spectrum of activity for substances using (PASS) online was performed to assess the overall biological potential of the organic molecule for the human organism. Based on the structures of organic compounds, the program provides simultaneous predictions of many types of biological activity. Through molecular structural analysis, the program provides a set of likely activities, giving various facets of biological action for a compound by means of an interpretation of Pa (probability “to be active”) and Pi (probability “to be inactive”) rates [22].

### 2.7. In Silico Study: Estimation of Pharmacokinetic Parameters and Toxicology

The pharmacokinetic parameters and theoretical toxicological ADMET (absorption, distribution, metabolism, excretion, and toxicity) were calculated using the admetSAR tool. The parameters were hematoencephalic barrier permeability, permeability Caco-2, absorption in the intestine, substrates and inhibitors of cytochrome enzymes, and inhibitors of renal cation transport. Through this tool, metabolism using certain cytochrome P450 enzymes was evaluated comparing whether the compounds were substrates for cytochromes CYP450 2D6, CYP450 3A4, and CYP450 2C9; whether they were inhibitors of cytochrome CYP450 1A2, CYP450 2C9, CYP450 2D6, CYP450 2C19, and CYP450 3A4; and whether there was cytochrome inhibition promiscuity [23].

### 2.8. Hemolysis Assay in Human Erythrocytes

A suspension of 0.5% of human erythrocytes in 0.9% NaCl was prepared. The solutions of isopropyl caffeate at different concentrations (10, 50, 100, 250, 500, and 1000 *μ*g/ml) were added to 2 ml of the erythrocyte suspension. The cell suspension was used as a negative control (0% hemolysis), and the erythrocyte suspension plus Triton X-100 was used as a positive control (100% hemolysis). The samples were incubated for 1 h under slow and constant stirring (100 rpm) at 22 ± 2°C. Afterwards, they were centrifuged at 2500 rpm for 5 min. Hemolysis was measured by spectrophotometry at a wavelength of 540 nm [24]. All experiments were performed in triplicate and expressed as an average plus or minus the standard error of the mean.

### 2.9. Evaluation of Osmotic Fragility of Human Erythrocytes

Solutions of isopropyl caffeate at concentrations of 10, 50, 100, 250, 500, and 1000 *μ*g/ml were incubated in 2 ml of a 0.5% suspension of erythrocytes for 1 hour at 25 ± 2°C. After this, the preparations were centrifuged at 2500 rpm for 5 minutes and the supernatant discarded. The erythrocytes were then resuspended in a hypotonic sodium chloride solution (0.24%) solution and shaken at 100 rpm for 20 minutes at 25 ± 2°C. The hemolysis was quantified by spectrophotometry at 540 nm [25]. A suspension of erythrocytes was used as a negative control (0% hemolysis), and a solution of erythrocytes in the presence of a 0.24% sodium chloride solution was used as a positive control (100% hemolysis). The experiments were performed in triplicate, and the results expressed as % hemolysis as compared to the positive control group.

### 2.10. Assessment of Oxidant and Antioxidant Potential in Human Erythrocytes in the Presence of Phenylhydrazine

To investigate the oxidizing potential, a 30% hemoglobin solution in PBS supplemented with glucose (200 mg/dl), pH 7.6 was prepared. Isopropyl caffeate (10, 100, 250, 500, and 1000 *μ*g/ml) was then added to 2 ml of erythrocyte suspension and incubated for 1 h under slow and steady stirring (100 rpm) at 25 ± 2°C. The samples were then centrifuged at 2500 rpm for 5 min, and the percentage of methemoglobin (MetHb) in relation to total hemoglobin (Hb) was quantified by spectrophotometry at 630 nm and 540 nm, respectively. The percentage of MetHb formed was compared to values obtained in the presence the phenylhydrazine (PH), a proven oxidizing agent.

To investigate the antioxidant potential, after the incubation period of 1 h, for the step described above, we added 1 mmol/l of phenylhydrazine. The solutions were aerated and kept under constant and slow agitation (100 rpm) for another 20 min to 25 ± 2°C. After this period, the samples were centrifuged at 2500 rpm for 5 min and diluted in PB, and the percentage of MetHb in relation to total Hb was quantified by spectrophotometry at 630 nm and 540 nm, respectively.

The percentage of MetHb formed was compared with the values obtained for vitamin C (1000 *μ*g/ml), a proven antioxidant agent. The experiments were performed in triplicate, and the results expressed as the methemoglobin formation percentage, in function of hemoglobin–MetHb (% Hb) [26].

### 2.11. Evaluation of the Antioxidant Potential in Human Erythrocytes in the Presence of Reactive Oxygen Species

The experiment was carried out in accordance with a study by Khalili et al. [27], with modifications. Isopropyl caffeate (10, 100, 250, 500, and 1000 mg/ml) was incubated with 2 ml of a 0.5% erythrocyte suspension in 0.9% NaCl. After 5 min of incubation, 0.5 ml of H_2_O_2_ (0.3%) was added. An erythrocyte suspension was used as negative control (0% cell hemolysis), an erythrocyte suspension in the presence of H_2_O_2_ (0.3%) as positive control (100% hemolysis), and an erythrocyte suspension in the presence of H_2_O_2_ (0.3%) and vitamin C (1000 *μ*g/ml) as the reference antioxidant. After 2 h, the samples were centrifuged at 2500 rpm for 10 min, and hemolysis was quantified by spectrophotometry at 540 nm [24].

### 2.12. Acute Oral Toxicity Test Procedures

The *in vivo* toxicological properties were investigated according to OECD Guideline 423 with modifications [28]. The control group was orally administered dilution vehicle. The group treated with isopropyl caffeate initially received the starting dose of 300 mg/kg, with subsequent repetition of the dose. Nonoccurrence of death directed the experiment to continue using the 2000 mg/kg dose, finalizing with another repetition. We used 3 Balb/c females per group, except in the control group (6 females). After administration of isopropyl caffeate, behavioral parameters were observed for signs of pharmacological effects on the central nervous system at 30, 60, 90, 120, 180, and 240 min [29]. Food and water consumption was monitored daily, and body weight was recorded on day 0 (before dosing), day 7, and day 14. After 14 days, the animals were sacrificed by administration of excess anesthetic (20 mg/kg xylazine and 500 mg/kg ketamine). Blood was removed for laboratory analysis of biochemical parameters. Organs were weighed (the heart, stomach, spleen, liver, and kidneys), with subsequent histopathological examinations. Also, through quantification of malondialdehyde and nitric oxide, evaluation of the hepatic antioxidant system was performed.

#### 2.12.1. Biochemical Parameters

Biochemical analyses were carried out using a ChemWell® automated biochemical analyzer (lab test) to determine the serum levels of glucose, urea, creatinine, cholesterol, aspartate aminotransferase (AST), alanine aminotransferase (ALT), and alkaline phosphatase.

#### 2.12.2. Determination of Malondialdehyde (MDA) Concentration

Lipid peroxidation was measured through levels of MDA [30], with modifications. The tissues were homogenized in KCl solution 1.15% (*m*/*v*) (1 g of tissue/5 ml) using a tissue grinder and then centrifuged at 1200 rpm for 10 minutes, at 0°C. After centrifugation, the supernatant was reacted with thiobarbituric acid (TBA 0.8%) in the presence of acetic acid (20%) and sodium dodecyl sulfate (SDS 8.1%), for 60 minutes at 100°C. After cooling, n-butanol was added to the reaction, the tubes were centrifuged, and the reading of the organic phase was performed in a microplate reader at a wavelength of 535 nm. The standard curve was constructed using 1,3-tetraethoxypropane.

#### 2.12.3. Measurement of Nitric Oxide (NO)

The nitrite concentration in the liver homogenate obtained was used as an index of nitric oxide production and was measured using the Griess reaction. Briefly, 50 *μ*l of each sample and 50 *μ*l of Griess reagent were placed in a 96-well microtiter plate, incubated at room temperature (22°C) for 10 min, while being protected from light. The absorbance was measured at a wavelength of 560 nm using a microplate reader, and the nitrite concentration was determined by comparing the sample absorbance to a standard curve for sodium nitrite. The experiments were performed in triplicate, and the results expressed in *μ*M [31].

#### 2.12.4. Anatomopathological and Histopathological Evaluation

After euthanasia, the organs were examined macroscopically; resection was made with consecutive weighing of the heart, stomach, liver, spleen, and kidneys (severed by sagittal incision). The organ indexes were calculated according to the formula: index = weight of component (mg)/animal's weight (g). The tissue sections of the organs excised (the stomach, liver, spleen, and kidneys), fixed in formaldehyde solution 10%, after 72 hours were resectioned for histopathological processing: dehydration with an increasing alcohol series (70 to 100%), diaphanization in xylol, impregnating, and inclusion in paraffin, according to the usual methods. The reagent fragments were sectioned with a thickness of 3.0 *μ*m, mounted on slides, and stained with hematoxylin and eosin and then examined under an optical microscope at 200 and 400x [32].

### 2.13. Statistical Analysis

Values are expressed as mean ± standard deviation (SD). Statistical significance between groups was determined using a one-way analysis of variance (ANOVA) followed by Dunnett's test, with *p* < 0.05 indicating significance. All statistical analyses were performed using GraphPad Prism 6.0 (GraphPad Software Inc., La Jolla, CA, USA).

## 3. Results

### 3.1. In Silico Tests: Molinspiration

Isopropyl caffeate was shown to be moderately active and likely produces its actions through physiological interaction as a GPCR ligand, an ion channel modulator, a nuclear receptor ligand, and enzyme inhibitor (Table 1). This result follows from a score of greater than 0.00, which is likely to have significant biological activities. Values between −0.50 and 0.00 represent moderated bioactivity. If the score is less than −0.50, the compound is presumed to be inactive [33, 34].

### 3.2. In Silico Tests: Pass Online

In this test, one may verify probabilities for isopropyl caffeate to be active (PA) with antieczematic, antihelmintic (nematodes), antihypercholesterolemic, antihypoxic, antimutagenic, antiprotozoal (*Leishmania*), antiseborrheic, antiseptic cytoprotective, free radical sequestrating, hepatoprotective, or lipid peroxidase-inhibiting properties (Table 2).

### 3.3. In Silico Tests: admetSAR

This test revealed that isopropyl caffeate has no theoretical toxicity, has good solubility, stability, and absorption, and does not suffer significant first pass metabolism in the liver or small intestine. However it is subject to active transportation, though the compound does not theoretically permeate the blood-brain barrier. Also, isopropyl caffeate was not classified as a substrate or as an inhibitor for the assessed CYP450 isoforms, possessing low promiscuity (Table 3).

### 3.4. *In Vitro* Hemolysis

**Figure 2:**
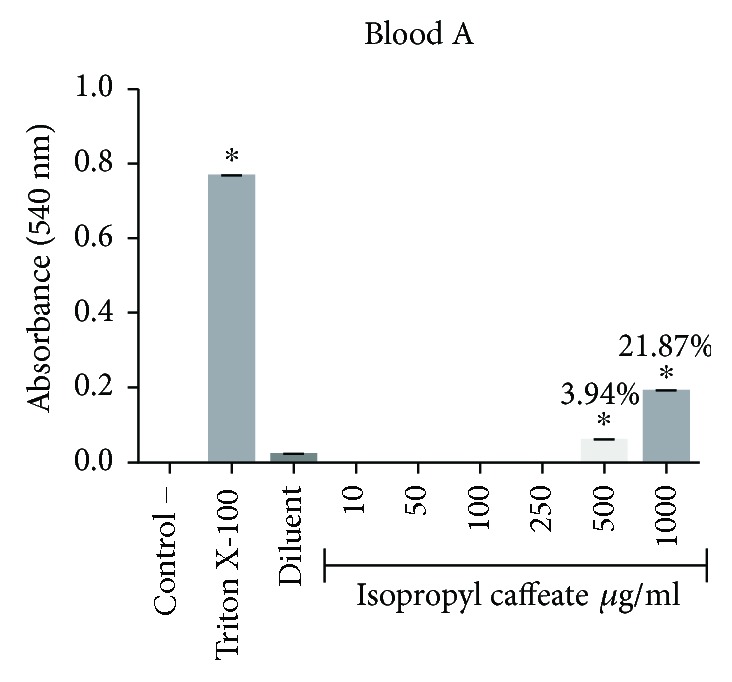
Hemolytic evaluation in type A erythrocytes, as induced by isopropyl caffeate. The results are expressed as mean ± SD analysis by ANOVA followed by Dunnett's test, ^∗^*p* < 0.05 (*n* = 3).

**Figure 3:**
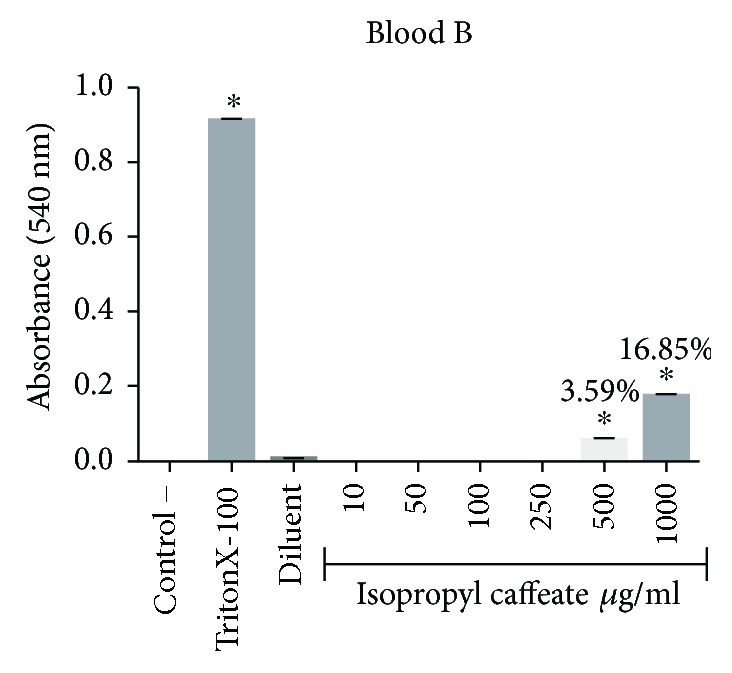
Hemolytic evaluation in type B erythrocytes, as induced by isopropyl caffeate. The results are expressed as mean ± SD analysis by ANOVA followed by Dunnett's test, ^∗^*p* < 0.05 (*n* = 3).

**Figure 4:**
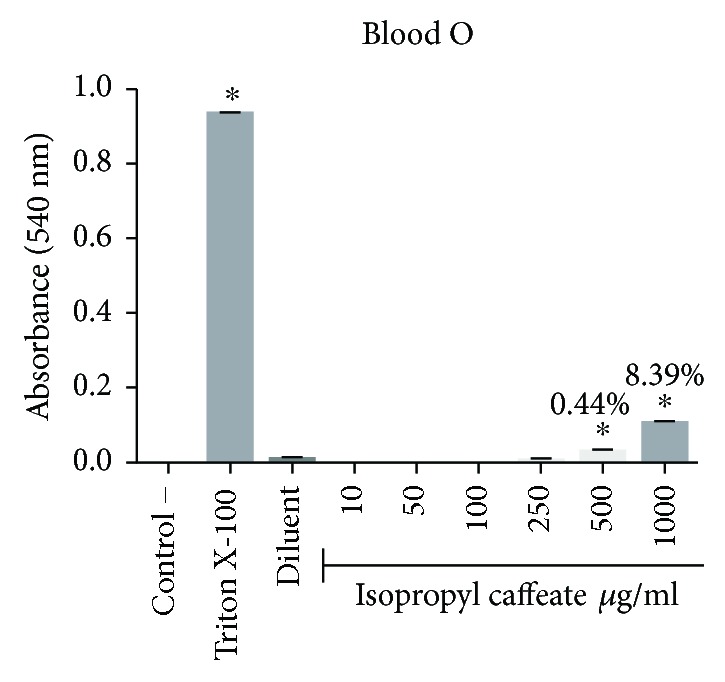
Hemolytic evaluation in type O erythrocytes, as induced by isopropyl caffeate. The results are expressed as mean ± SD analysis by ANOVA followed by Dunnett's test, ^∗^*p* < 0.05 (*n* = 3).

**Figure 5:**
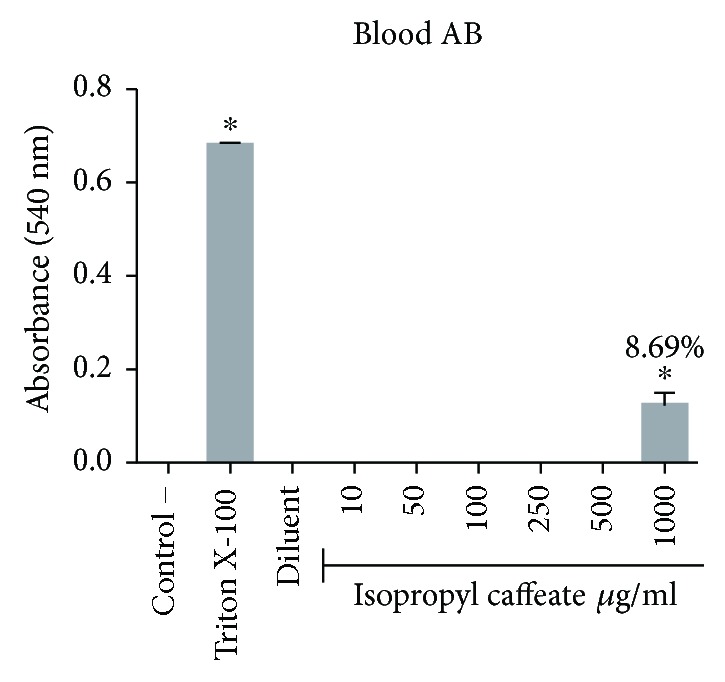
Hemolytic evaluation in type AB erythrocytes, as induced by isopropyl caffeate. The results are expressed as mean ± SD analysis by ANOVA followed by Dunnett's test, ^∗^*p* < 0.05 (*n* = 3).

The isopropyl caffeate was only able to promote hemolysis at concentrations of 500 *μ*g/ml and 1000 *μ*g/ml, for the blood types A, B, and O. For the blood type AB, only the concentration of 1000 *μ*g/ml was able to promote hemolysis when compared to the negative control. The percentage of hemolysis observed at a concentration of 500 *μ*g/ml ranged from 0.44% to 3.94%, and at the concentration of 1000 *μ*g/ml, the range between the blood types was from 8.39% to 21.87%, (Figures 2–5).

### 3.5. *In Vitro* Osmotic Fragility

**Figure 6:**
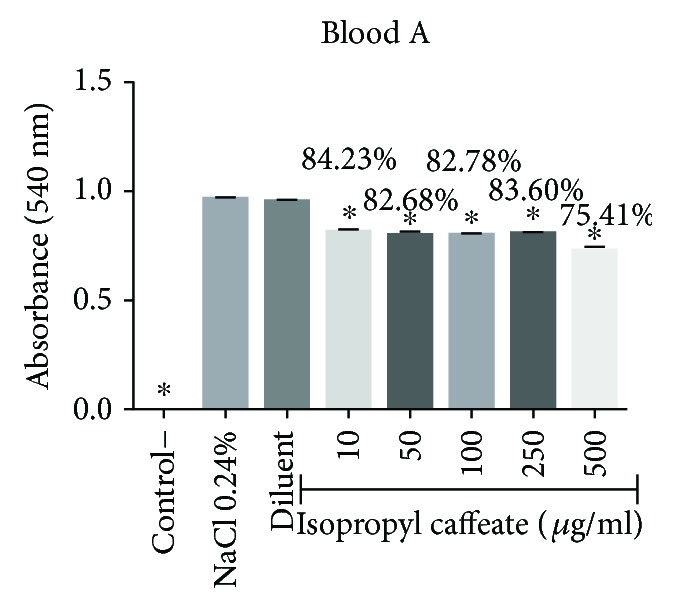
Antihemolytic evaluation in erythrocytes type A, induced by isopropyl caffeate, when in a hypotonic solution (NaCl 0.24%). The results are expressed as mean ± SD analysis by ANOVA followed by post-Dunnett's test, ^∗^*p* < 0.05 (*n* = 3).

**Figure 7:**
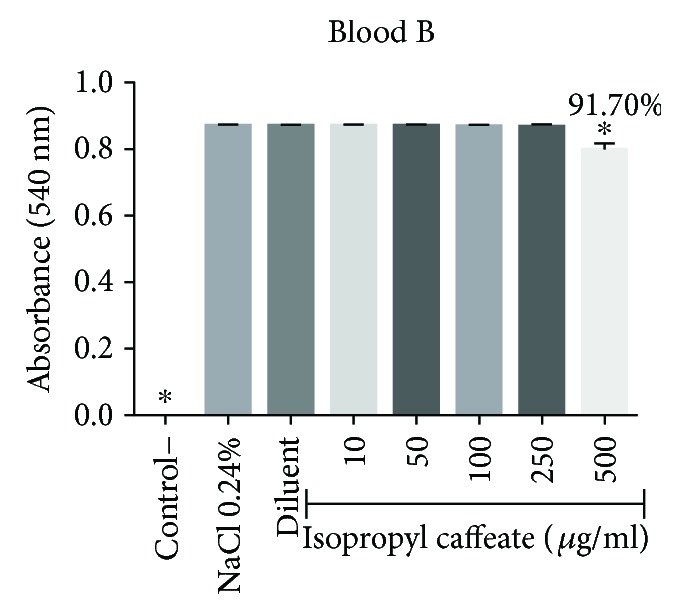
Antihemolytic evaluation in type B erythrocytes, as induced by isopropyl caffeate, when in a hypotonic solution (NaCl 0.24%). The results are expressed as mean ± SD analysis by ANOVA followed by Dunnett's test, ^∗^*p* < 0.05 (*n* = 3).

**Figure 8:**
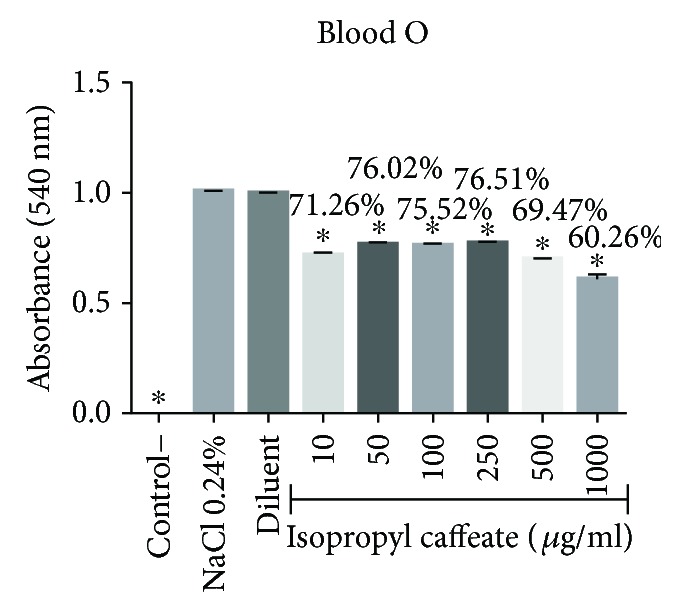
Antihemolytic evaluation in type O erythrocytes, as induced by isopropyl caffeate, when in a hypotonic solution (NaCl 0.24%). The results are expressed as mean ± SD analysis by ANOVA followed by Dunnett's test, ^∗^*p* < 0.05 (*n* = 3).

**Figure 9:**
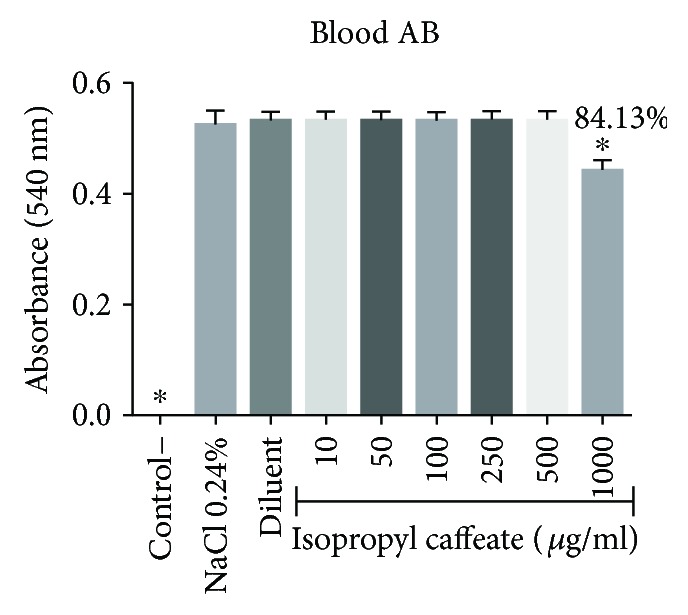
Antihemolytic evaluation in type AB erythrocytes, induced by isopropyl caffeate, when in a hypotonic solution (NaCl 0.24%). The results are expressed as mean ± SD analysis by ANOVA followed by Dunnett's test, ^∗^*p* < 0.05 (*n* = 3).

Isopropyl caffeate, at all concentrations tested, was capable of inhibiting hemolysis caused by osmotic differences selectively in blood types A and O and can be seen in Figures 6–9.

### 3.6. *In Vitro* Oxidant and Antioxidant in the Presence of Phenylhydrazine

**Figure 10:**
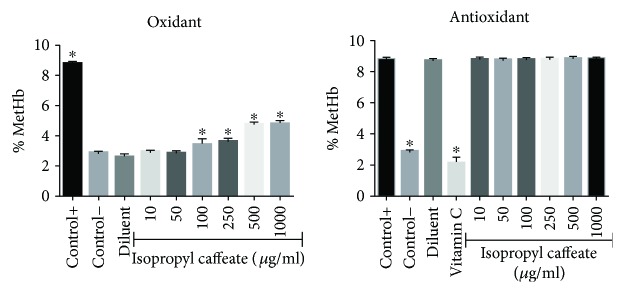
Oxidizing and antioxidant effects of isopropyl caffeate in human erythrocytes. The results are expressed as a percentage of average of formation of methemoglobin (MetHb) compared to the negative control group (oxidant test) and a positive control (antioxidant test). The results are expressed as mean ± SD analysis by ANOVA followed by Dunnett's test, ^∗^*p* < 0.05 (*n* = 3).

Figure 10 shows that the isopropyl caffeate was unable to oxidize the cells at concentrations of 10, 50, and 100 *μ*g/ml, when compared to the negative control group (Hb), and no concentration was able to oxidize the cells when compared to the positive control group (Hb + Ph), characterizing no significant oxidant power in this substance. For antioxidant activity in the presence of phenylhydrazine, no reduction was observed in the percentage of methemoglobin formation in function of hemoglobin (MetHb/% Hb) as compared to the positive control group (Hb + Ph).

### 3.7. *In Vitro* Antioxidant in the Presence of Reactive Oxygen Species

**Figure 11:**
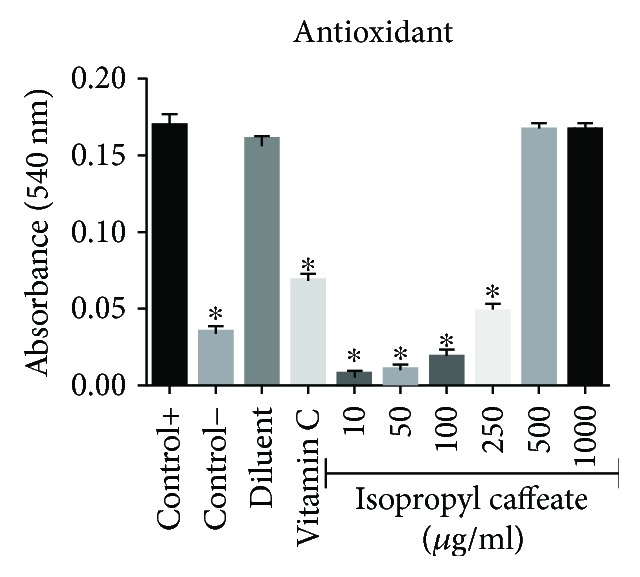
Antioxidant activity of isopropyl caffeate against hemolysis induced by hydrogen peroxide in blood type O. Results are expressed as a percentage of average in comparison to the positive control group (Hb + H_2_O_2_). The results are expressed as mean ± SD analysis by ANOVA followed by Dunnett's test, ^∗^*p* < 0.05 (*n* = 3).

The ability of isopropyl caffeate at concentrations of 10, 50, 100, and 250 *μ*g/ml to sequestrate ROS was observed. A reduction of hemolysis induced by hydrogen peroxide (H_2_O_2_) was verified when compared to the positive control group (Hb + H_2_O_2_). It is important to stress that isopropyl caffeate presented greater antioxidant power than vitamin C (Figure 11).

### 3.8. *In Vivo* Acute Toxicity: Behavioral Changes, Consumption of Water and Food and Weight Gain

In assessment of acute toxicity at the dose of 300 mg/kg, there were no deaths. At this dose, depressive signs in central nervous system (CNS), such as reduction of hyperactivity, sedation, loss of atrial reflex, and response to touch, were identified. Also, signals related to the autonomic nervous system (ANS) were identified, such as constipation and decreased muscle tone. There were also decreases in self-cleaning and climbing behavior.

At a dose of 2000 mg/kg, we observed similarities in the behavior of animals that had been treated with a dose of 300 mg/kg. However, not having verified greater intensity in these effects, we cannot correlate dosage with acute toxic effect. Intense piloerection was observed, which is a sign of CNS stimulation. These behavioral changes disappeared after 24 h for both administrated doses. Also, isopropyl caffeate presented analgesia and induced catatonia at two of the treatment doses. However, this was not observed in all animals of the treated group; only a few were in fact brought to this effect which persisted for 3 hours. After eight days of administration, two animals died during the dose repetition process, at 2000 mg/kg.

According to the “Globally Harmonized System of Classification” (GHS), isopropyl caffeate was considered in category 4 (DL_50_> 300–2000), according to guide number 423 of the OECD [28], and this category warning is necessary [35]; the LD_50_ was estimated at 2000 mg/kg. This data corroborates the theoretical LD_50_ demonstrated by the admetSAR software that yielded 1.8847 mol/kg.

There was a statistically significant change (being the elevation of two compatible parameters) in the consumption of water and food and an increase in the weight gain of these same animals during the first week (animals treated with isopropyl caffeate at 300 mg/kg). For the animals treated at a dose of 2000 mg/kg, there was weight loss during the first week (Table 4).

### 3.9. *In Vivo* Acute Toxicity: Biochemical Parameters

We observed possible liver protective activity, since ALT and AST levels were lower in relation to the negative control group in the two treatment dosages. At the dose of 300 mg/kg, levels of urea decreased; this gives evidence of renal protection as well (Table 5).

### 3.10. *In Vivo* Acute Toxicity: Determination of MDA and NO

**Figure 12:**
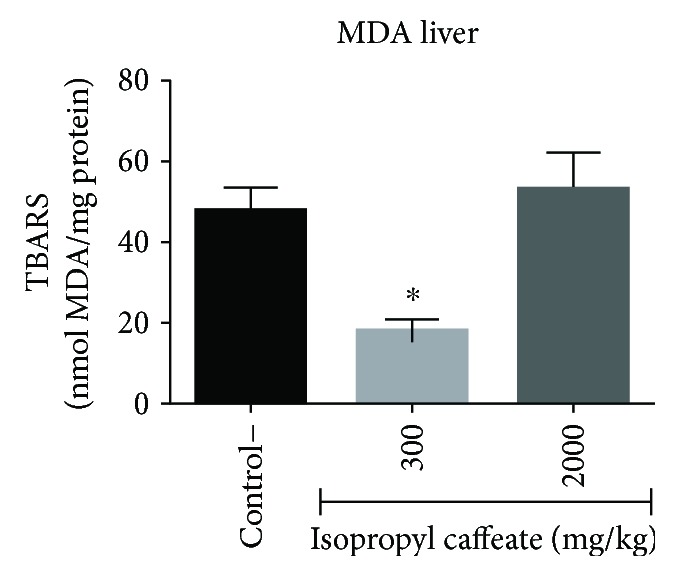
Dosage of malondialdehyde (MDA) of liver homogenate of Balb/c females submitted to administration of isopropyl caffeate. The results are expressed as mean ± SD analysis by ANOVA followed by Dunnett's test, ^∗^*p* < 0.05 (control and dose of 300 mg/kg, *n* = 6/dose of 2000 mg/kg, *n* = 4).

**Figure 13:**
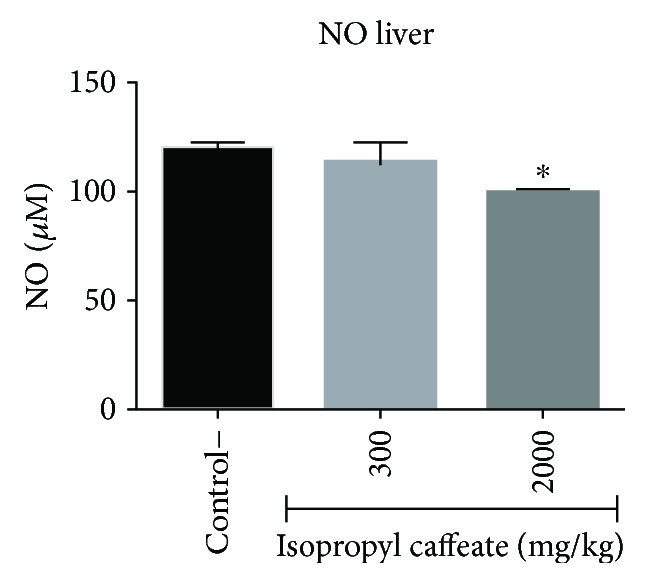
Determination of NO in liver homogenate of Balb/c females submitted to administration of isopropyl caffeate. The results are expressed as mean ± SD analysis by ANOVA followed by Dunnett's test, ^∗^*p* < 0.05 (control and dose of 300 mg/kg, *n* = 6/dose of 2000 mg/kg, *n* = 4).

Again, we note potential protective action in the liver; in the animals treated at a dose of 300 mg/kg, there was a statistically significant decrease in the production of MDA and consequent decreases in lipid peroxidation and oxidative stress. A decrease in the production of NO in the animals treated with a dose of 2000 mg/kg was verified. These results corroborate the PASS online software for the probability of being active with hepatoprotection (Figures 12 and 13).

### 3.11. *In Vivo* Acute Toxicity: Anatomopathological and Histopathological

**Figure 14:**
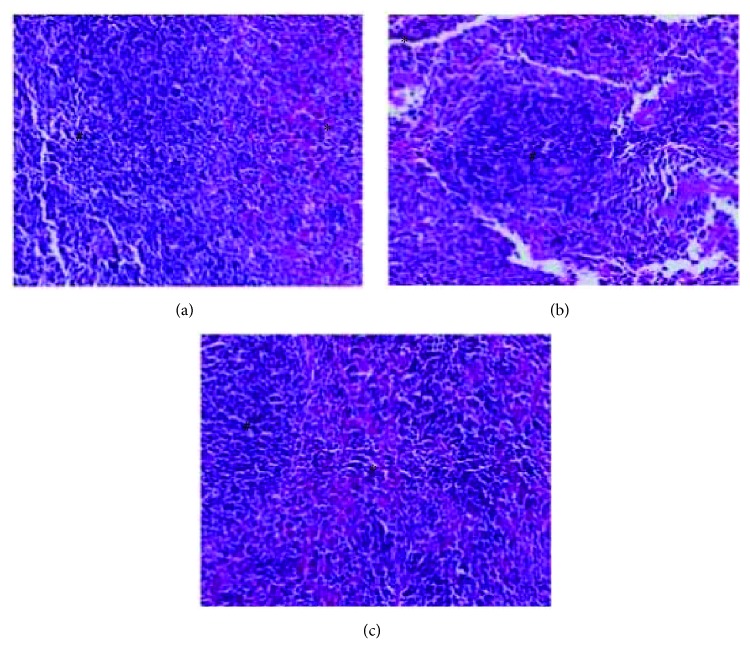
Spleen of treated Balb/c females V.O., stained with hematoxylin and eosin (increase 200x), there was no sign of toxicity in the spleen. Asterisks appoint red pulp and white flesh (#) without special histological features. (a) Control group vehicle. (b) Group treated with a dose of 300 mg/kg. (c) Group treated with a dose of 2000 mg/kg.

**Figure 15:**
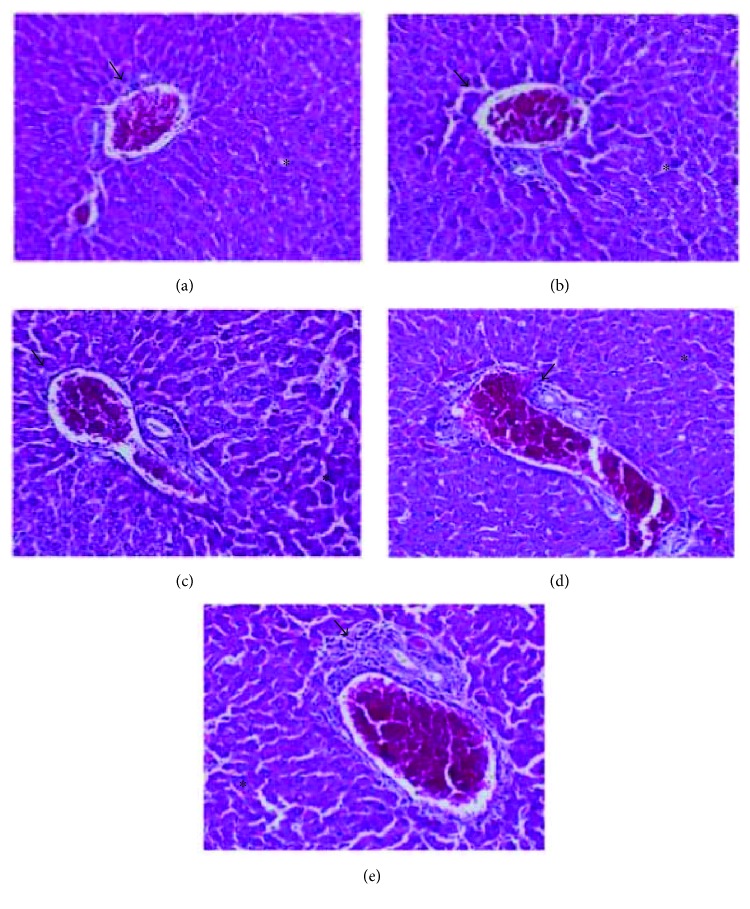
Liver of treated Balb/c females V.O., stained with hematoxylin and eosin (increase 200x), there was no sign of toxicity in the liver. The arrows appoint portal spaces and the asterisks appoint normal hepatocytes. (a) Control group vehicle. (b) Group treated with a dose of 300 mg/kg. (c) Group treated with a dose of 2000 mg/kg. (d) Animal treated with a dose of 2000 mg/kg with little inflammatory infiltrate portal (portal spaces (arrow) with mild chronic inflammatory infiltration). (e) Animal treated with a dose of 2000 mg/kg (portal spaces (arrow) with moderate chronic inflammatory infiltration).

**Figure 16:**
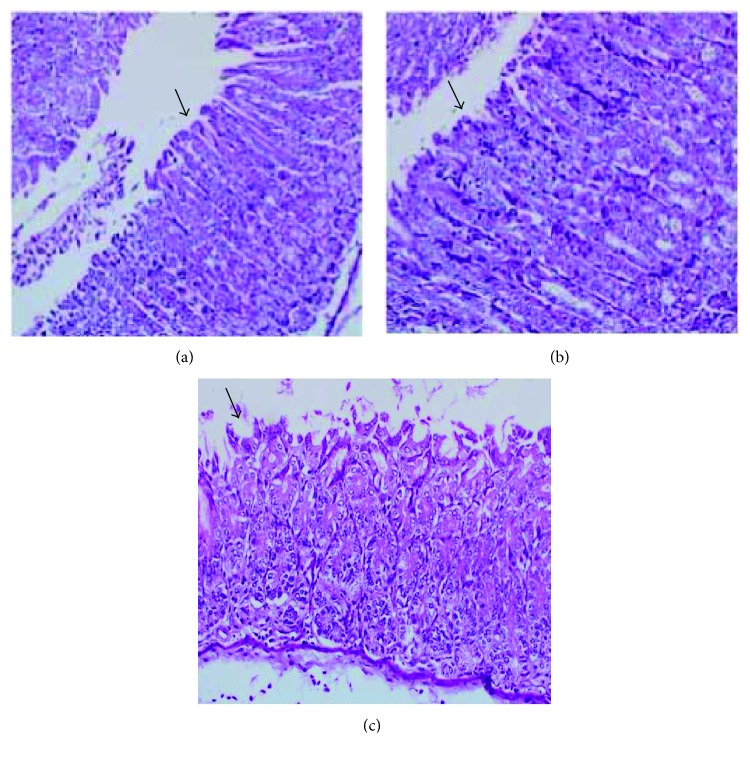
Stomach of treated Balb/c females V.O., stained with hematoxylin and eosin (increase 200x), there was no sign of toxicity in the stomach. The arrow appoints normal gastric mucosa. (a) Control group vehicle. (b) Group treated with a dose at 300 mg/kg. (c) Group treated with a dose of 2000 mg/kg.

In assessing organ weights, the spleen and lungs showed small significant variations as compared to the control group (Table 6), yet with no changes in structural integrity (Figure 14). The architecture of the renal parenchyma and circulatory system also presented no changes. The liver presented chronic inflammatory portal infiltration of mild to moderate intensity in two animals, however, not affecting the structure or normal architecture of this organ. There was no serious sign of substance toxicity (Figure 15). However, the stomach showed minimal edema and unspecific chronic inflammatory infiltration in the chorion, without inflammatory-destructive crypt activity or signs of gastric lesions induced by the drug (Figure 16).

## 4. Discussion

One way to predict the toxicity and pharmacokinetics of substances is through in silico tests, which assess the toxicity of theoretical compounds and aid in the choice of potentially promising molecules [11, 12, 36]. In silico tests revealed that isopropyl caffeate has no theoretical toxicity (Table 3) and has probable activity against several diseases which are directly linked to oxidation (Table 2).

From the in silico results, determination of cytotoxicity was performed using red blood cells since they contain high concentrations of polyunsaturated fatty acids, molecular oxygen, and ions linked in the ferrous state, making the cells highly vulnerable to reactions involving free radicals and susceptible to lipid peroxidation in cell membranes, which contributes to occurrences of hemolysis [37–39].

Another mechanism that may be related to hemolysis is erythrocyte apoptosis. Although erythrocytes do not have mitochondria that signal for this process, if there is exposure to ceramide or sphingosine, or an increasing concentration of cytosolic Ca^2+^/([Ca^2+^]_i_), oxidative stress and depletion of energy occur, the stimulation that leads to the erythrocyte apoptosis [40].

Cytotoxicity was also a measure of osmotic fragility, since cells become more osmotically fragile when there are changes in membrane lipids and proteins causing a reduction in the membrane integrity [41, 42]. Changes in osmotic fragility may be related to the inhibition of enzyme activity and depletion of antioxidant defenses [39, 43].

The osmotic fragility results indicate that isopropyl caffeate causes significant changes in membrane proteins and in the structure of human erythrocyte lipids for the blood types B and AB. However, the increase in osmotic fragility observed for blood type AB may be due to interaction between isopropyl caffeate and aglutinogen B, since in blood type A, we observed a decrease in osmotic fragility, which was not verified in blood type B.

The phenylhydrazine is a powerful oxidizing agent that causes damage to hemoglobin through formation of hemichrome. In the heme group, a hydroxyl radical is added to hydroxilate a methene/phenyldiazene bridge (phenyldiazene is a derivative of phenylhydrazine) and binds to the heme group in 6th position, forming oxyhemoglobin [44]. As seen in Figure 10, isopropyl caffeate was not capable of inhibiting this mechanism.

Hydrogen peroxide is a key metabolite in oxidative stress [18, 45]. Phenolic compounds act as antioxidants due to aromatic ring hydroxyl substituent reactivity, and the mechanism of antioxidant activity is elimination of the radical [2, 46]. Wang et al. [6] demonstrated the antioxidant activity of caffeic acid and its derivatives using the DPPH test; the compounds were considered potent antioxidants, presenting greater reducing of DPPH than vitamin C. Isopropyl caffeate presented better antioxidant activity than caffeic acid. The esterification of caffeic acid had a positive effect, increasing the antioxidant stability of the compound.

Potential toxicity of a substance refers to its ability to cause some imbalance in the body with which it comes into contact [36, 47]. The analgesia presented by isopropyl caffeate suggests an antinociceptive profile due to a reduction in mechanical stimuli response [48, 49]. This result corroborates Buzzi et al. [7], who evaluated the antinociceptive properties of isopropyl caffeate and verified antinociceptive effect similar to certain therapeutic drugs such as acetylsalicylic acid, acetaminophen, and morphine. In substances that prevent both pain and inflammation, a certain correlation between antinociceptive and antioxidant properties has been demonstrated [50].

Drugs or drug therapies can readily cause catatonia [51, 52]. The test result from the central nervous system for this substance is contrary to the theoretical admetSAR software results, which predicted that isopropyl caffeate should not pass the blood-brain barrier. The piloerection observed is a parameter that occurs in rodents as a result of fear, disease, or pharmacological stress [53–55]. In addition to behavioral variations, the toxicity of a compound can be demonstrated by means of changes in animal weight development, reduction in the consumption of water and food, and changes in the excretion of urine and feces [56, 57].

The weight gain of the animals may be affected by difficulty in digestion and absorption of food and nutrients, an indication of possible toxic effect [58–60]. However, the results are in agreement with OECD recommendations for body weight variation [28], which not exceed 20% variation in average weight (Table 4).

To evaluate hepatic and renal function after administration of isopropyl caffeate, investigation of biochemical parameters was performed. Such protective action may be linked to the antioxidant activity of isopropyl caffeate, since free radicals are responsible for diseases involving liver damage and kidney failure [4, 15] (Table 5).

Pang et al. [61] demonstrated caffeic acid's mechanism of protective action *in vivo* against hepatotoxicity induced by acetaminophen against liver cells. Caffeic acid is able to induce activation of Nrf2, an important antioxidant transcription factor, reducing the expression of inhibitory protein Keap1 and blocking Nrf2 binding with Keap1, with consequent increased antioxidant enzyme (NQO1 and HO-1), expression which prevents oxidative lesions in the liver as induced by acetaminophen.

Another way to investigate liver function is through quantification of malondialdehyde (MDA) and nitric oxide (NO). Excessive production of MDA has been associated with various pathological states, since it is a secondary product formed during lipid peroxidation [62, 63]. An elevated production of NO decreases regulation of cytochrome P450; it triggers suppression of hepatic proteins and the synthesis of DNA and thus induces apoptosis and necrosis [64, 65].

The liver, kidney, heart, spleen, and lungs are the organs most often affected by metabolic reactions caused by toxic agents [59]. Acute hepatotoxicity may be characterized as injury similar to hepatitis involving all areas of the gland, with hepatocellular ballooning, acidophilic hepatocyte degeneration, apoptotic bodies, small foci of necrosis, and discrete portal inflammation [66]. Thus, we note that isopropyl caffeate did not present elevated toxicity (corroborating the data calculated by the software) and also demonstrated possible hepatoprotection.

In conclusion, we note that isopropyl caffeate presents a range of pharmacological activities while demonstrating low cytotoxicity and *in vivo* toxicity, it protects cells in the presence of hypotonic solution, and does not compromise membrane structure, yet interferes in erythrocytic membrane functions. In this study, we reaffirm the antioxidant activity of isopropyl caffeate already presented in other studies as well as report other demonstrated effects such as elimination of free radicals. Isopropyl caffeate thus constitutes a promising option for the development of future drugs and may increase the available therapeutic arsenal.

## Figures and Tables

**Table 1:** Prediction of bioactivities calculated in software Molinspiration, for isopropyl caffeate.

Substance	GPCR ligand	Ion channel modulator	Kinase inhibitor	Nuclear receptor ligand	Protease inhibitor	Enzyme inhibitor
Isopropyl caffeate	−0.44	0.25	−0.66	−0.01	−0.58	−0.09

**Table 2:** Suggestions indicating biological activities for isopropyl caffeate; analysis by PASS online tool.

PA	PI	Activity
0.521	0.017	Anticarcinogenic
0.603	0.084	Antieczematic
0.545	0.024	Antifungal
0.743	0.003	Antihelmintic (nematodes)
0.611	0.013	Antihypercholesterolemic
0.875	0.003	Antihypoxic
0.547	0.044	Anti-inflammatory
0.564	0.074	Anti-ischemic, cerebral
0.751	0.005	Antimutagenic
0.538	0.005	Antioxidant
0.557	0.010	Antiparasitic
0.651	0.011	Antiprotozoal (*Leishmania*)
0.556	0.023	Antipruritic
0.594	0.013	Antipruritic, allergic
0.554	0.015	Antipsoriatic
0.648	0.047	Antiseborrheic
0.534	0.024	Antisecretory
0.756	0.005	Antiseptic
0.577	0.018	Antithrombotic
0.584	0.012	Antiulcerative
0.532	0.019	Antiviral drug (influenza)
0.618	0.024	Cytoprotectant
0.659	0.005	Free radical scavenger
0.639	0.010	Hepatoprotectant
0.767	0.004	Lipid peroxidase inhibitor

**Table 3:** Sorting properties ADMET, calculated in software admetSAR, for isopropyl caffeate.

Model	Isopropyl caffeate
*Absorption*	
Hematoencephalic barrier	BBB−
Intestinal absorption man	HIA+
Permeability to Caco-2	Caco-2+
Substrate of glycoprotein-P	S
Inhibitor of glycoprotein-P	NI
Renal transport of organic cations	NI
*Distribution and metabolism*	
Substrate CYP450 2C9	NS
Substrate CYP450 2D6	NS
Substrate CYP450 3A4	NS
Inhibitor CYP450 1A2	NI
Inhibitor CYP450 2C9	NI
Inhibitor CYP450 2D6	NI
Inhibitor CYP450 2C19	NI
Inhibitor CYP450 3A4	NI
CYP inhibitor promiscuity	Low
*The excretion and toxicity*	
HERG	IF
Toxicity in Ames test	NT
Carcinogen	NC
Biodegradation	Prone to degradation
Acute oral toxicity	III
*ADMET profile prediction*—*regression*	
Aqueous solubility	−2.7631 (LogS)
Permeability to Caco-2	0.2988 (LogPapp, cm/s)
*Toxicity*	
Acute oral toxicity (rats)	1.8847 (LD50, mol/kg)

+ (permeates); NI: does not inhibit; NS: not substrate; S: substrate; I: inhibits; IF: weak inhibitor; NT: not toxic; NC: not a carcinogen; HERG: *gene related* to *light*-a-*go*-*go human.*

**Table 4:** Effects of acute isopropyl caffeate treatment on the consumption of water and food and weight gain of treated female mice.

Groups	Dose (mg/kg)	Water (ml)	Food (g)	Weight gain (day 7)	Weight gain (day 14)
Control	—	6.190 ± 0.31	4.697 ± 0.14	1.100 ± 0.46	1.100 ± 0.46
Treated	300	7.673 ± 0.31^∗^	5.536 ± 0.19^∗^	2.397 ± 0.64^∗^	0.772 ± 0.10
Treated	2000	6.884 ± 0.35	4.306 ± 0.25	−1.093 ± 0.38^∗^	0.375 ± 0.28

The results are expressed as mean ± SD analysis by ANOVA followed by Dunnett's test, ^∗^*p* < 0.05 (control and treated at 300 mg/kg, *n* = 6/treated at 2000 mg/kg, *n* = 6 in the first week and *n* = 4 in the second week).

**Table 5:** Effects of acute isopropyl caffeate treatment on biochemical parameters of female mice.

Groups	Dose (mg/kg)	AST (U/I)	ALT (U/I)	Alkaline phosphatase (U/I)	Serum creatinine (mg/dl)	Urea (mg/dl)	Glucose (mg/dl)	Cholesterol (mg/dl)
Control	—	322.4 ± 12.69	144.5 ± 12.55	116.2 ± 4.09	0.31 ± 0.02	44.00 ± 2.26	188.3 ± 5.58	91.00 ± 2.89
Treated	300	177.2 ± 17.58^∗^	62.00 ± 5.90^∗^	125.5 ± 9.19	0.36 ± 0.01^∗^	34.40 ± 2.38^∗^	201.5 ± 9.35	88.83 ± 3.29
Treated	2000	166.5 ± 20.90^∗^	55.25 ± 4.05^∗^	103.3 ± 11.79	0.31 ± 0.01	42.67 ± 2.33	188.3 ± 11.78	97.33 ± 5.49

The results are expressed as mean ± SD analysis by ANOVA followed by post-Dunnett's test, ^∗^*p* < 0.05 (control and dose of 300 mg/kg *n* = 6/dose of 2000 mg/kg *n* = 4).

**Table 6:** Effects of acute isopropyl caffeate treatment on the content of the organs of female mice.

Groups	Dose (mg/kg)	Heart contents (mg/g)	Lung index (mg/g)	Stomach contents (mg/g)	Liver contents (mg/g)	Spleen index (Mg/g)	Left kidney index (mg/g)	Right kidney index (mg/g)
Control	—	5.06 ± 0.12	21.818 ± 1.03	10.89 ± 0.47	51.86 ± 1.19	7.04 ± 0.42	7.16 ± 0.22	7.07 ± 0.29
Treated	300	4.98 ± 0.15	5 ± 3.18	10.74 ± 0.68	55.99 ± 1.64	7.01 ± 0.44	7.66 ± 0.18	7.67 ± 0.05
Treated	2000	5.6 ± 0.37	14.9 ± 2.42^∗^	10.37 ± 0.55	53.69 ± 2.87	9.48 ± 0.92^∗^	7.24 ± 0.16	7.37 ± 0.18

The results are expressed as mean ± SD analysis by ANOVA followed by post-Dunnett's test, ^∗^*p* < 0.05 (*N* = 6).
